# Supplementary material for: In situ atomic-scale observation of oxidation and decomposition processes in nanocrystalline alloys
Source: Nat Commun. 2018 Mar 5;9:946. doi: 10.1038/s41467-018-03288-8 (PMC5838172; doi:10.1038/s41467-018-03288-8)
Supplement: Supplementary file 1 — Supplementary Information [file 41467_2018_3288_MOESM1_ESM.pdf]

Supplementary Information

In-situ atomic-scale observation of oxidation and decomposition processes in nanocrystalline alloys

Guo et al.

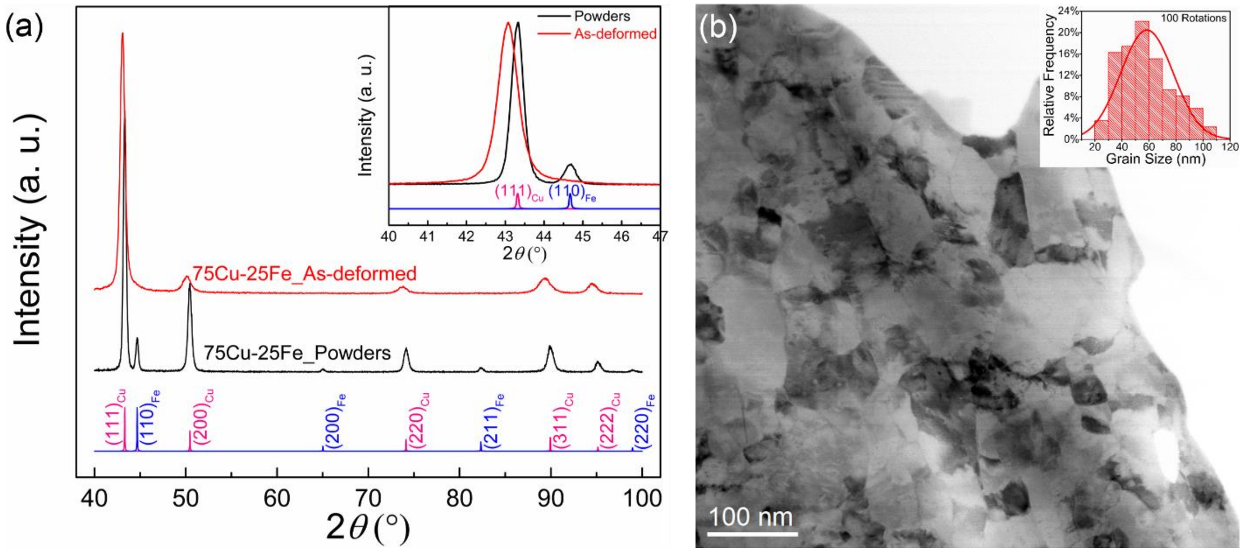

Supplementary Figure 1: (**a**) XRD comparison between compacted powders and HPT-deformed sample of 75Cu-25Fe with 100 rotations. The enlarged profiles focusing on $(111)_{Cu}$ and $(110)_{Fe}$ peaks are displayed as inset at top-right corner. (**b**) Bright-field image and grain size statistics of as-deformed 75Cu-25Fe sample with 100 rotations.

Supplementary Note 1: Microstructure characterization of as-deformed 75Cu-25Fe alloys.

Supplementary Fig. 1a shows the XRD profiles of compacted powders and as-deformed sample of 75Cu-25Fe. It is clearly shown that the blended powders were severely deformed and formed a single *fcc* phase after 100 rotations deformation. The calculated *fcc* lattice parameter increases from 3.613 ± 0.001 Å for pure Cu powders to 3.634 ± 0.001 Å due to the 25 at.%Fe incorporation. Supplementary Fig. 1b shows the bright-field image of as-deformed 75Cu-25Fe sample with 100 rotations, and the statistics of grain size, which indicates a mean grain size of about 58 nm.

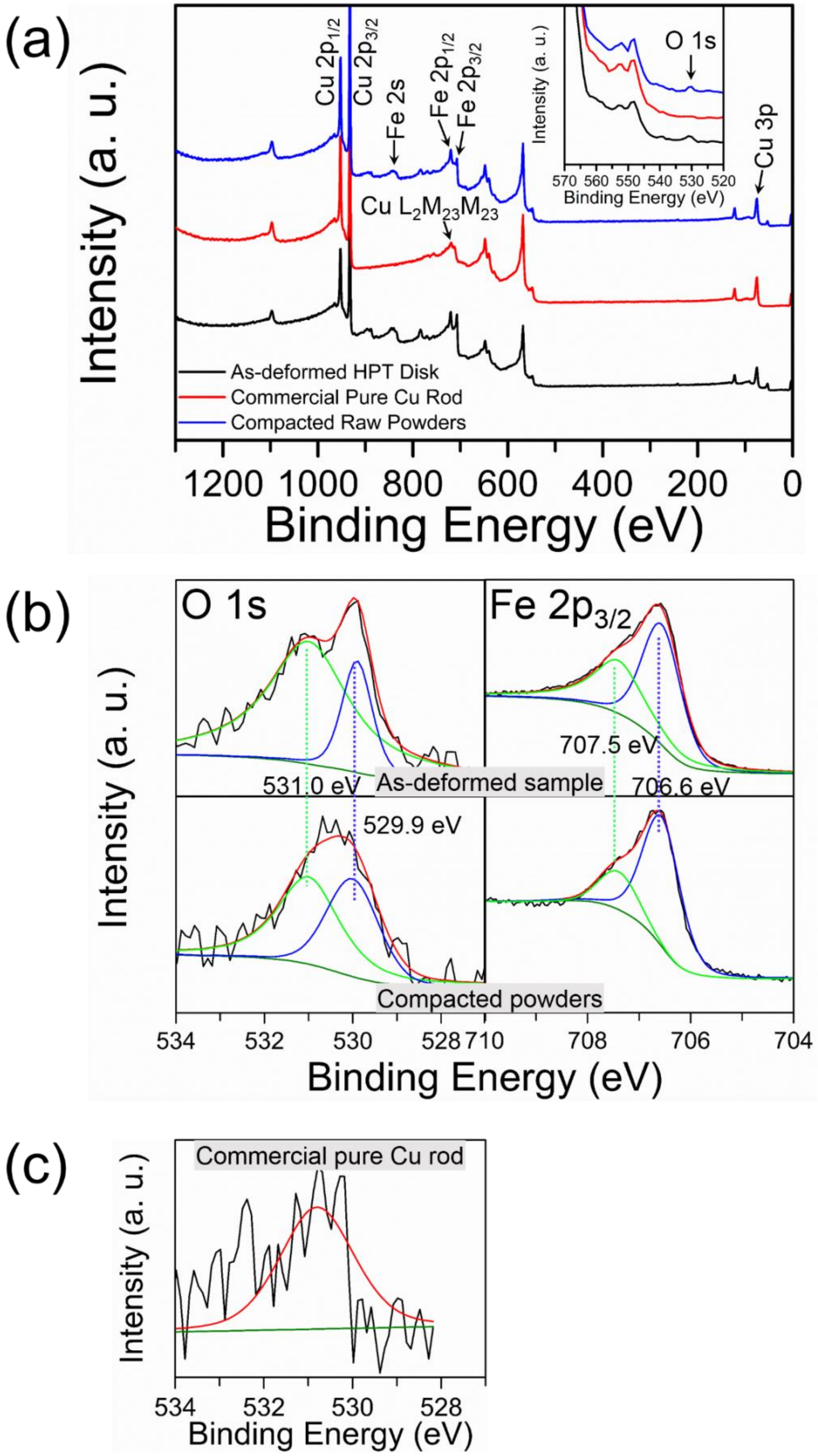
(a)
Intensity (a. u.)
Cu 2p1/2
Cu 2p3/2
Fe 2s
Fe 2p1/2
Fe 2p3/2
Cu L2M23M23
Cu 3p
O 1s
As-deformed HPT Disk
Commercial Pure Cu Rod
Compacted Raw Powders
Binding Energy (eV)
(b)
O 1s
Fe 2p3/2
707.5 eV
531.0 eV
As-deformed sample
706.6 eV
529.9 eV
Compacted powders
Binding Energy (eV)
(c)
Commercial pure Cu rod
Intensity (a. u.)
Binding Energy (eV)

Supplementary Figure 2: (**a**) XPS spectra of 75Cu-25Fe as-deformed HPT sample (black spectrum), commercial pure Cu rod (red spectrum) and 75Cu-25Fe compacted raw powders (blue spectrum). (**b**) Fine measurements of O 1s and Fe $2p_{3/2}$ peaks of 75Cu-25Fe as-deformed HPT sample and compacted raw powders. (**c**) Fine measurement of O 1s peak of commercial pure Cu rod.

Supplementary Table 1: Constituents of 75Cu-25Fe as-deformed sample, commercial pure Cu rod and 75Cu-25Fe compacted raw powders calculated from respective XPS spectra.

Samples	O (at.%)	Cu (at.%)	Fe (at.%)
As-deformed HPT disk	3.6	74.9	21.5
Commercial pure Cu rod	< 1.0	> 99.0	-
Compacted raw powders	3.0	73.3	23.7

Supplementary Note 2: XPS measurements of constituents of as-deformed samples and comparison samples.

Oxygen contents in the initial raw powders, as-deformed HPT sample and a piece of commercial pure Cu material (nominal purity of 99.99%, used as a reference) were systematically measured by X-ray photoelectron spectroscopy (XPS, ESCALAB 250Xi, Thermo Fisher Scientific, Waltham, USA). We have taken a series of special measures to remove the surface oxide layers before transfer the samples to the XPS chamber. All sample surfaces were fully

polished in a media of ethyl alcohol and then transferred to XPS chamber immediately, which was followed by Ar ion sputtering with ion energy of 3 keV for 5 minutes to completely remove the possible surface oxide layers. We would emphasize that all samples were kept in ethyl alcohol after polishing, and the operation time of transfer from ethyl alcohol to XPS chamber was controlled to a minimum of about a few seconds. All three samples mentioned above were transferred into the chamber at the same time.

Supplementary Fig. 2a shows the spectra in a full range of 0 – 1300 eV. Some typical peaks of Cu and Fe are indexed on the profiles. To clearly check the O 1s peaks, the spectra within the region of 520 – 570 eV are enlarged and shown as an inset in Supplementary Fig. 2a. While O 1s peaks can be seen clearly for the 75Cu-25Fe as-deformed sample and the compacted powders, almost no signal can be observed for the commercial pure Cu rod sample at binding energy of 530 – 532 eV. The comparative study by XPS provides the direct evidence of oxygen presence in powder samples.

Supplementary Fig. 2b shows the fine measurements of O 1s and Fe $2p_{3/2}$ peaks of 75Cu-25Fe as-deformed HPT sample and compacted raw powders respectively, and Supplementary Fig. 2c shows the fine measurement of O 1s peak of commercial pure Cu rod. For the Fe $2p_{3/2}$ peak, it can be seen that each well resolved Fe $2p_{3/2}$ spectrum shows multiplet splitting with second component shifting to a higher energy by 0.9 eV from the main peak. The position of main peak at 706.6 eV and the typical asymmetric peak shape containing a 0.9 eV higher component at 707.5 eV, both attest the Fe atoms are substantially in zero-valent states. If we compare the O 1s peak shown in Supplementary Fig. 2c with the O 1s peaks of 75Cu-25Fe as-deformed HPT sample and compacted raw powders, we can see the signal-to-noise ratio of the O 1s peak of commercial Cu rod is very low and the peak is nearly negligible, which means probably only

very tiny amount of oxygen exists in the pure Cu rod. Meanwhile, it proves that our measurements are effective without introducing adsorbed oxygen.

To evaluate the content of oxygen in each sample, peaks of O 1s, Cu $2p_{3/2}$ and Fe $2p_{3/2}$ were selected to quantify the integral areas. Supplementary Table 1 displays the quantification result of the contents of O, Cu and Fe in 75Cu-25Fe as-deformed sample, commercial pure Cu and 75Cu-25Fe compacted powders, taking into account relative sensitivity factors of 2.881 (O), 16.73 (Cu) and 10.7 (Fe). It can be seen that the powder samples contain a level of about 3 at.% oxygen inside material while for commercial pure Cu the oxygen content is less than 1 at.%. It should be emphasized that for the fine scan spectrum of commercial pure Cu, the signal-to-noise ratio of O 1s peak is quite low and it is hard to calculate the integral area of this peak accurately, so the given value here of 1 at.% is very likely overestimated.

The measured oxygen content in 75Cu-25Fe as-deformed disk here is 3.6 at.%. Actually we have also carried out independent measurements for another two as-deformed samples, the oxygen contents in these two samples are 3.4 at.% and 3.3 at.% respectively. So it can be assured that the oxygen content in the HPT as-deformed samples is about 3.5 at.%. As for the reason why the measured oxygen content in the compacted powders is 3.0 at.%, a little bit lower than the value in the HPT-deformed sample, it might be that the pores formed during compaction are segmented and the embedded oxygen react with metal elements during continuous deformation.

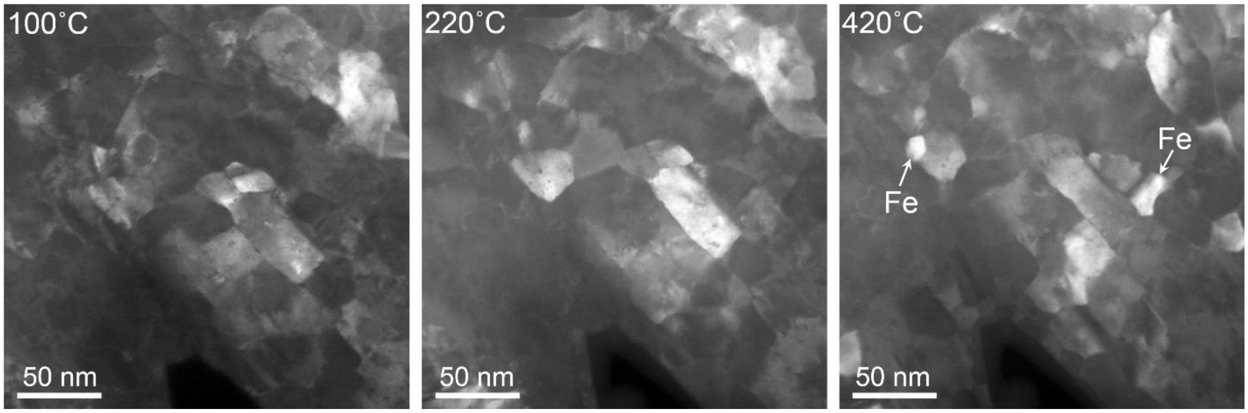

Supplementary Figure 3: *In-situ* observation of microstructure change of 75Cu-25Fe annealed at different temperatures with ADF-STEM image.

Supplementary Note 3: *In-situ* observation of microstructure change of 75Cu-25Fe alloy.

Supplementary Fig. 3 shows the ADF-STEM images of 75Cu-25Fe alloy taken at different temperatures. Intuitively, the grains hardly coarsen as the temperature increases. After the sample was annealed at 420 °C, we carried out EDXS line scans on the newly-grown grains, which were determined to be Fe grains as indicated by white arrows in the right-hand side image.

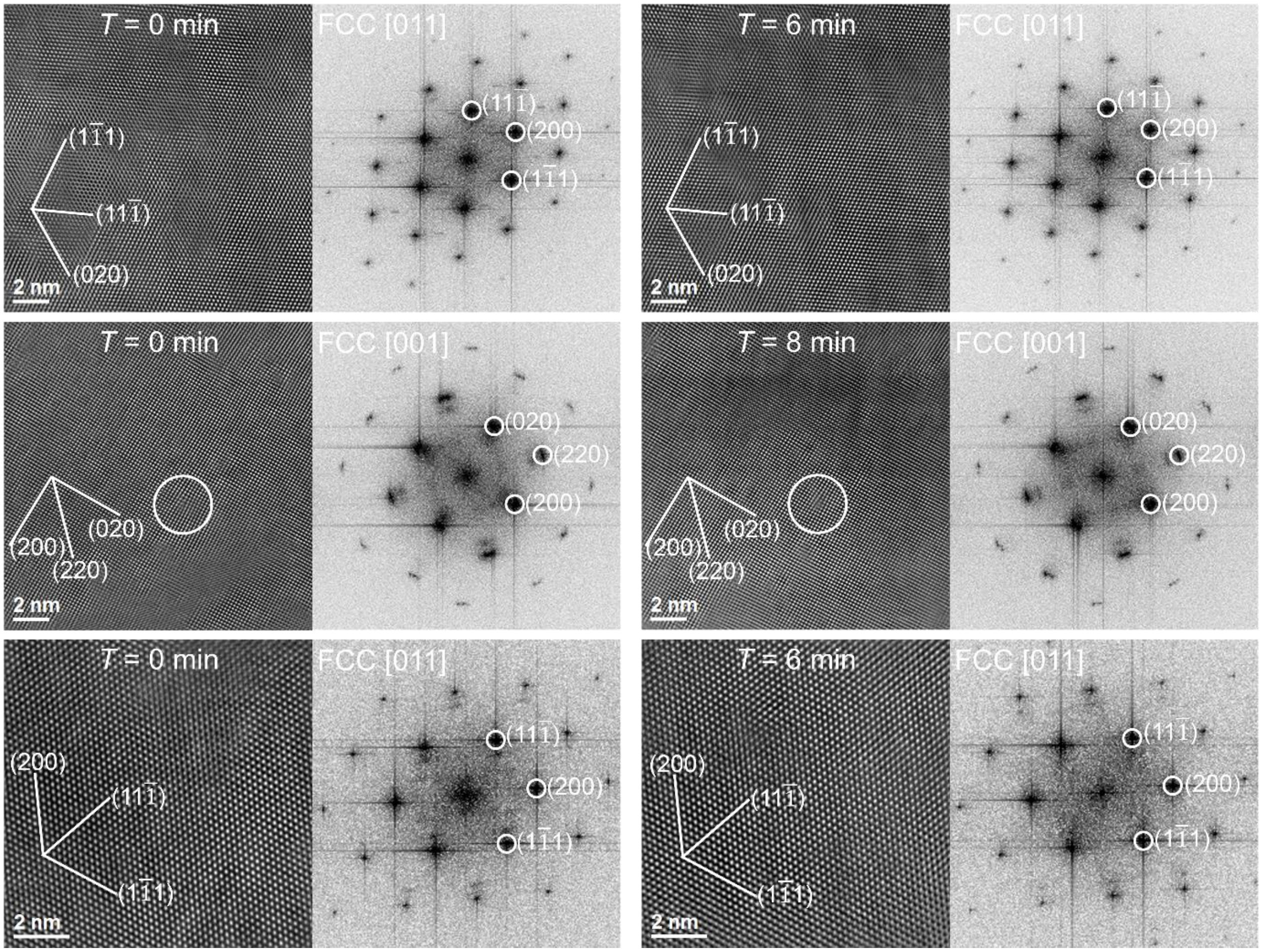

Supplementary Figure 4: Electron beam irradiation test on three different grains (each row represents one grain) with irradiation time of 6 – 8 min.

Supplementary Note 4: Electron beam irradiation test on different grains.

To verify that oxidation and decomposition are not caused by e-beam irradiation, a series of comparative experiments were carried out with a sample exposed to electron beam under the same dose conditions as those in the *in-situ* experiment being performed. The corresponding images under different acquisition time are shown in Supplementary Fig. 4. Electron beam was diverged to a specific size as same as the large fluorescent screen during irradiation. The result

shows that after exposure for several minutes, no obvious change has happened. Despite of the complexity of electron beam effects, our systematic experiments proved that beam effects under current conditions are negligible.

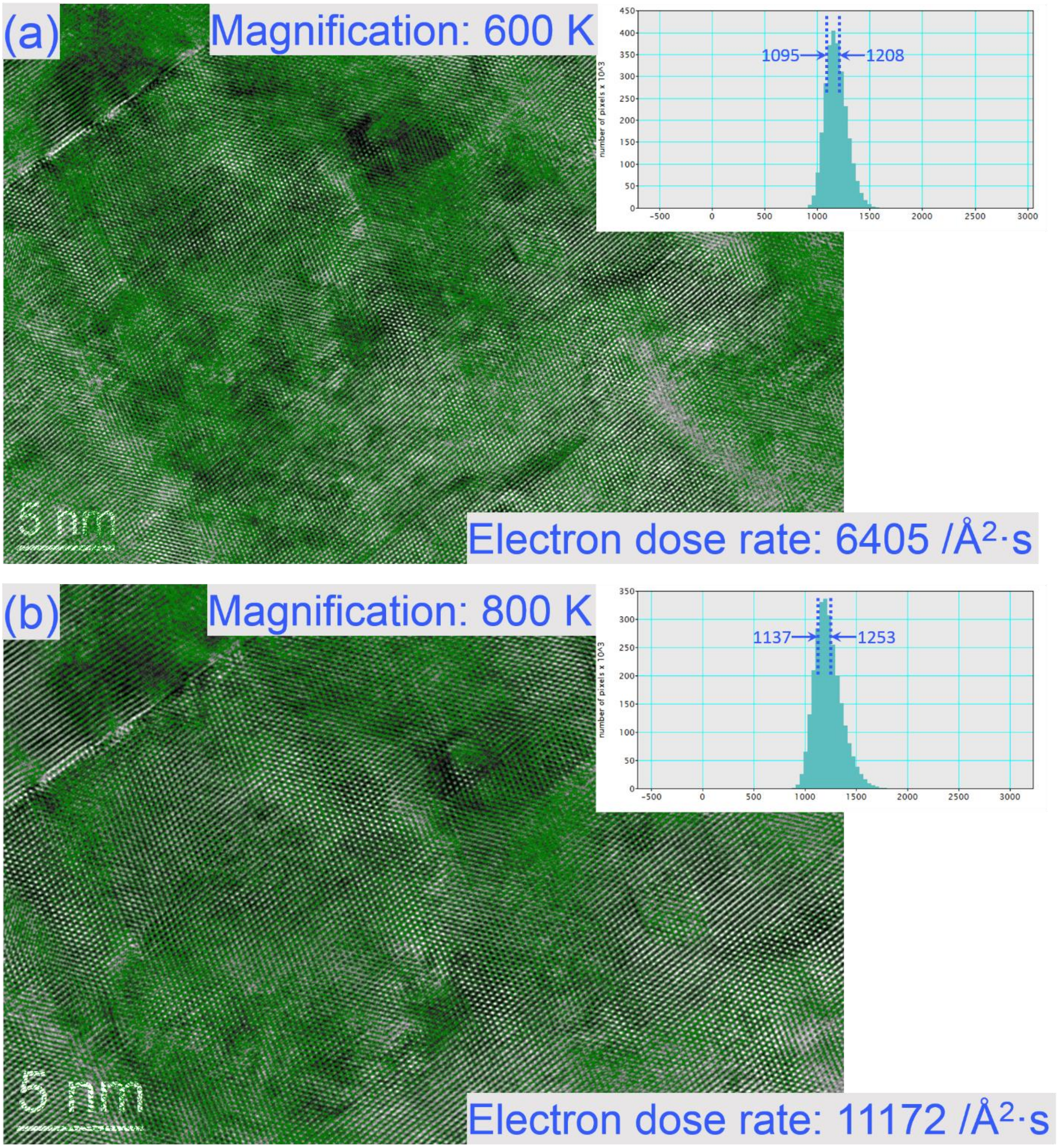

Supplementary Figure 5: Electron dose rate analyses of HRTEM images of the same area taken at magnification of (**a**) 600K and (**b**) 800K. The insets are distributions of the number of pixels versus the counts.

Supplementary Note 5: Electron dose rate analyses of HRTEM images.

In addition, we have quantified the electron dose rate on two images with magnifications of 600K and 800K, respectively, recorded on the same area as shown in Supplementary Fig. 5. The two insets are distributions of the number of pixels versus the counts. The green coverage areas shown in two HRTEM images correspond to the majorities of pixel intensities within a region shown in two insets. With the exposure time of 1 s and beam spreading to fit to the size of fluorescent screen, the electron dose rate for the image taken at magnification of 600K is 6405 $/\text{Å}^2\cdot s$ while it is 11172 $/\text{Å}^2\cdot s$ for image with the magnification of 800K. Under the imaging conditions as mentioned and configurations of CCD applied, the converted current density is about 68.8 pA/cm^2 for magnification of 600K and 69.2 pA/cm^2 for magnification of 800K respectively. Moreover, because the TEM sample is very thin, the heat dissipation usually proceeds very fast.

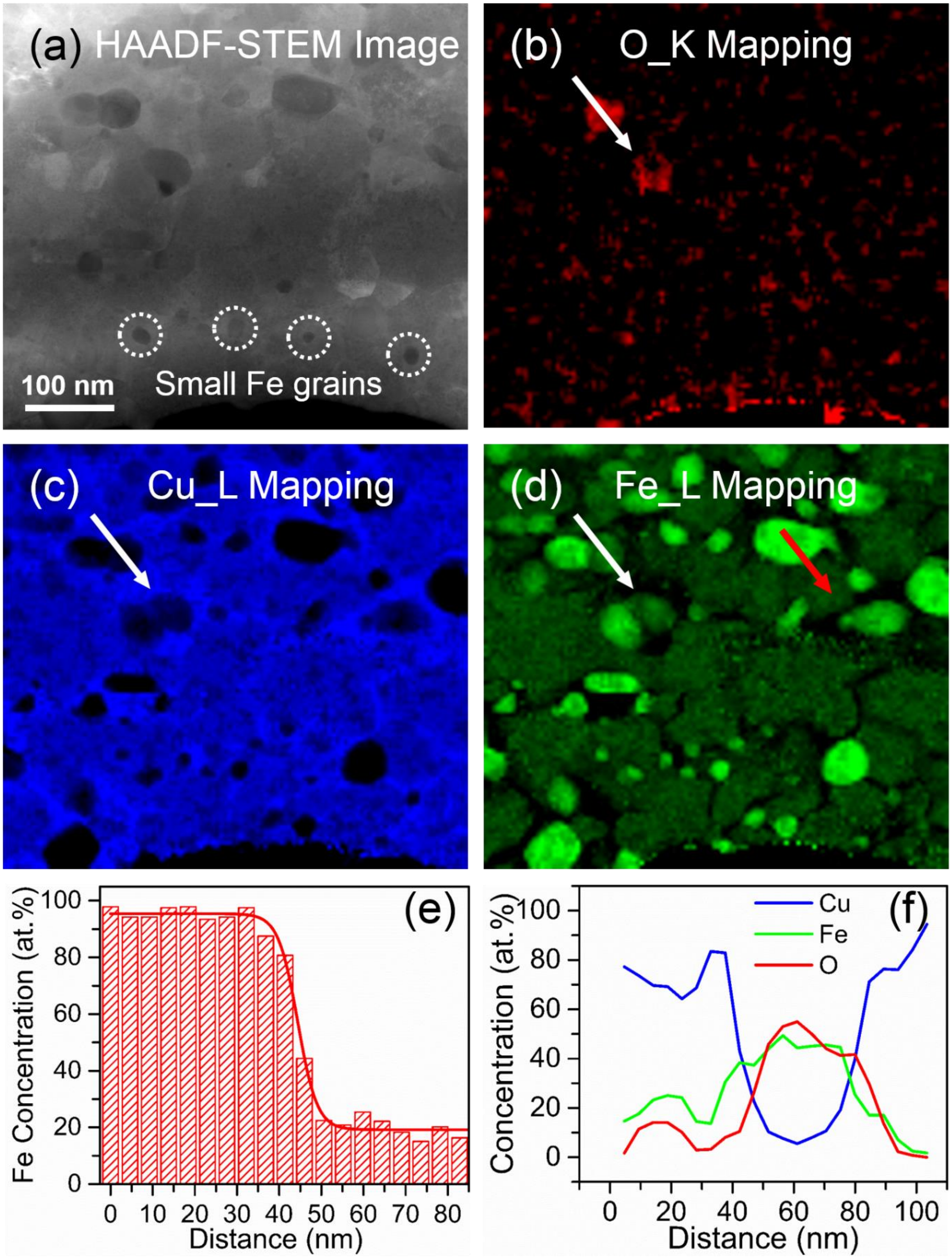

(a) HAADF-STEM Image
100 nm
Small Fe grains
(b) O_K Mapping
(c) Cu_L Mapping
(d) Fe_L Mapping
(e)
Fe Concentration (at.%)
Distance (nm)
0
10
20
30
40
50
60
70
80
100
(f)
Cu
Fe
O
Concentration (at.%)
Distance (nm)
0
20
40
60
80
100

Supplementary Figure 6: EELS elemental mapping of *ex-situ* annealed sample at 420 °C. (**a**) HAADF-STEM image. (**b**) O_K mapping. (**c**) Cu_L mapping. (**d**) Fe_L mapping. (**e**) Fe concentration histogram obtained from line scan along red arrow displayed in (**d**). (**f**) Cu, Fe and O concentration profiles extracted from line scan of the same position indicated by the white arrows in (**b-d**).

Supplementary Note 6: EELS elemental mapping of *ex-situ* annealed sample at 420 °C.

EELS mapping of the *ex-situ* annealed sample was implemented to confirm the EELS mapping results from the *in-situ* heating sample. Supplementary Fig. 6 shows the EELS elemental mapping of the sample *ex-situ* annealed at 420 °C. From the Fe_L mapping in Supplementary Fig. 6d, it can be seen that Fe grains grown due to the decomposition usually have rounded corner shapes. The morphologies of Fe grains are almost the same as the *in-situ* annealing results, with grain size of 20 – 50 nm. By comparing the Fe_L mapping, it can be seen that the dark areas in Supplementary Fig. 6a correspond to the Fe grains as marked with white circles. Supplementary Fig. 6b shows the O_K mapping, from which our conclusion on oxides formation during annealing with dimensions from several nanometers to tens of nanometers is proved. Besides some oxide precipitates present at the grain boundaries, many small oxide clusters formed inside the grains as observed by HRTEM images. The Fe concentration histogram shown in Supplementary Fig. 6e was extracted along a red arrow in Supplementary Fig. 6d from a pure Fe grain to the Cu matrix. It can be seen that after *ex-situ* annealing at 420 °C, the residual Fe in Cu matrix amounts to 18 – 20 at.% which is consistent with the result reported in Fig. 3c in the main text. Supplementary Fig. 6f shows line profiles crossing a Fe oxide particle formed within the sample as indicated by the white arrows in Supplementary Fig. 6b-d. It is clearly shown that the

Fe and O concentrations reach maxima while Cu concentration is at a minimum, which is a strong indication that Fe oxide forms within the grain instead of on the surface of the sample.

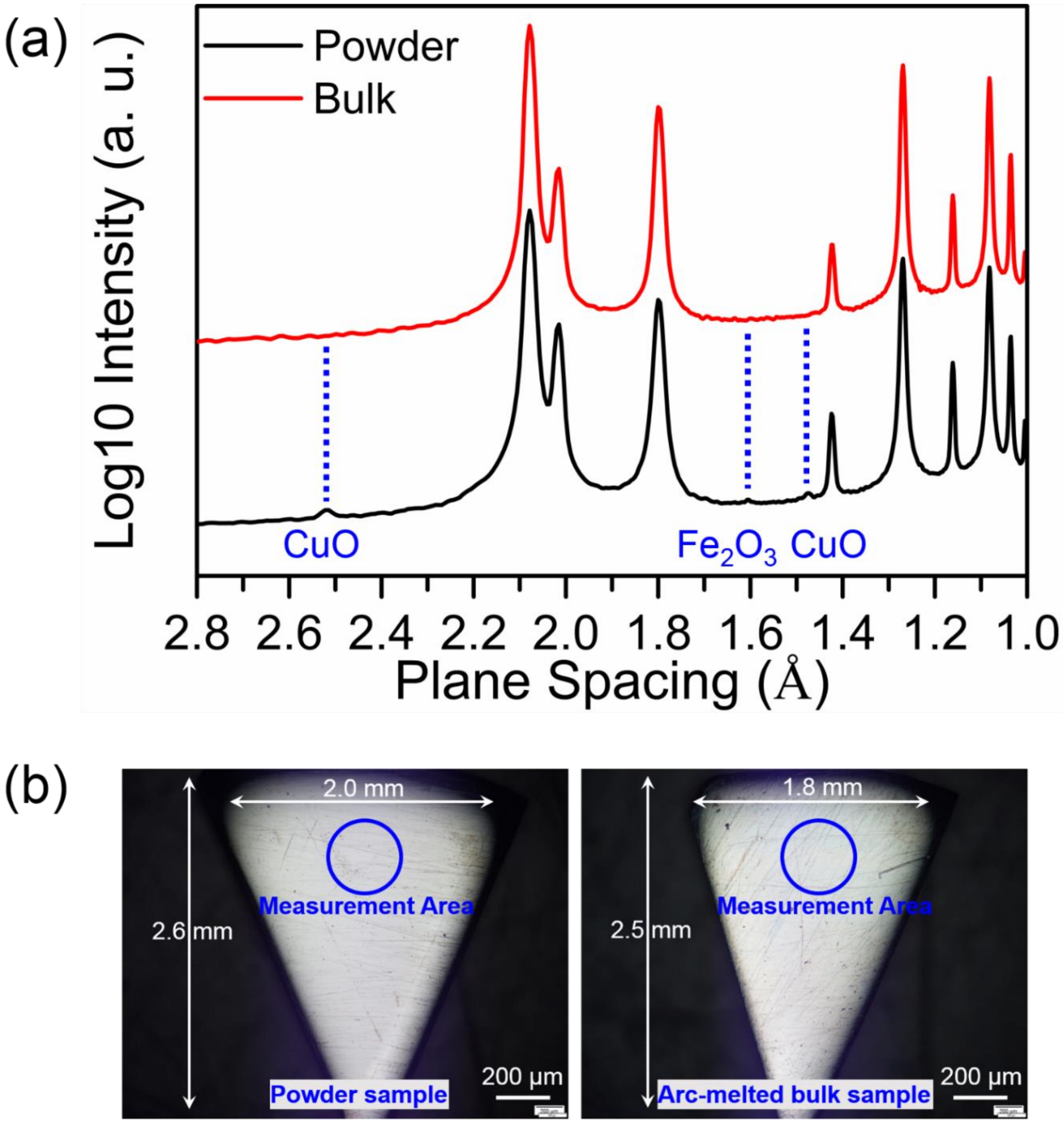

Supplementary Figure 7: (**a**) Synchrotron X-ray diffraction profiles of *ex-situ* annealed 75Cu-25Fe samples at 420 °C, which were deformed from powders and arc-melted bulk respectively.

(**b**) Images of powder and arc-melted bulk samples with blue circles indicating the measurement areas.

Supplementary Note 7: Synchrotron XRD measurements of 75Cu-25Fe samples deformed from powders and arc-melted bulk respectively.

To verify that the oxides formed inside materials after annealing, we measured the *ex-situ* annealed samples using synchrotron X-ray diffraction which has an extremely powerful energy to generate peaks even for tiny amounts of components. Synchrotron X-ray diffraction experiments were performed at the PETRA III P07 beamline at the DESY Photon Science facility (Hamburg, Germany). First, we melted a reference sample with the same composition of 75Cu-25Fe using the high purity commercial Cu and Fe rods (nominal purity: 99.99%). Because large pieces of Cu and Fe rods were used in arc-melting process, the potential influences of the surface oxides could be effectively reduced. Second, the powder sample and arc-melted sample with composition of 75Cu-25Fe were deformed by HPT to the same strains, followed by *ex-situ* annealing in Ar atmosphere at 420 °C at the same time. Then these two samples were cut and polished to the same shape and thickness. Synchrotron X-ray diffraction measurements were implemented on these two samples at the same conditions. Supplementary Fig. 7a shows the synchrotron profiles of *ex-situ* annealed 75Cu-25Fe samples at 420 ˚C, which were deformed from powders and arc-melted bulk respectively. Supplementary Fig. 7b shows the images of powder and arc-melted bulk samples with blue circles indicating the XRD measurement areas. From the diffraction profiles, we can see that except all Cu and Fe peaks locating at almost the same positions, for the profile of the powder sample, some extra peaks appear at the left side of $(111)_{Cu}$ peak as well as between $(200)_{Cu}$ and $(200)_{Fe}$ peaks, which fit to planes of CuO and Fe_2O_3 very well. The obvious

peak with spacing of 2.529 Å is indexed as $(002)_{CuO}$, and the peaks at positions of 1.603 Å and 1.475 Å can be indexed as $(120)_{CuO}/(122)_{Fe2O3}$ and $(124)_{Fe2O3}$ respectively. We used the arc-melted sample with the same composition as a reference, and the oxides can be only detected for the powder sample. So the synchrotron X-ray diffraction measurements provide a strong evidence that the oxides formed inside the sample after annealing. The detailed results and discussion of the differences between powder sample and arc-melted bulk sample will be given in our next paper.

Supplementary Note 8: DFT Calculations.

Vienna Ab-initio Simulation Package (VASP) was employed to calculate the formation enthalpies of CuO and Fe_2O_3. The projector augmented wave (PAW) method[1] was used to treat the interaction between ion and core electrons. The valence electrons were described using the local density approximation (LDA) and the generalized gradient approximation (GGA) with the exchange-correlation functional of Perdew, Burke and Enzerhof (PBE)[2]. Considering the strong correlation effects between transition metal elements and oxygen, Hubbard U model[3] was also employed. Here in our calculations, for Cu and Fe, U = 3 eV. Convergence tests indicated that 600 eV was a suitable cutoff energy for the PAW potential to obtain sufficient precision in the oxide systems. Brillouin-zone gridding was performed using the Monkhorst-Pack method[4,5] with 23×23×23 (Cu), 21×21×21 (*fcc*-structured Fe), 15×21×13 (CuO) and 17×17×3 (Fe_2O_3) k-point meshes, which were sufficient for structural optimization and static calculation. Energy of oxygen was calculated within a box of 12 Å×12 Å×12 Å. Spin polarization was considered in the Fe and Fe_2O_3 calculations. Using the optimal parameters, the calculated formation enthalpy

changes converge to better than 0.1 meV/atom. The results are shown in Supplementary Table 2, which are in reasonable agreements with previous results [6].

Supplementary Table 2: Calculated formation enthalpies of different oxides (eV/atom).

	**LDA**	**LDA+U**	**GGA**	**GGA+U**
**Cu_2O**	-0.703	-0.767	-0.599	-0.666
**CuO**	-1.091	-1.086	-0.873	-0.874
**Fe_2O_3**	-1.947	-1.980	-1.523	-1.563
**FeO**	-1.257	-1.745	-1.171	-1.576

Supplementary References

1. Kresse, G. & Joubert, D. From ultrasoft pseudopotentials to the projector augmented-wave method. *Phys*. *Rev*. *B* **59**, 1758–1775 (1999).

2. Perdew, J. P., Burke, K. & Ernzerhof, M. Generalized gradient approximation made simple. *Phys*. *Rev*. *Lett*. **77**, 3865–3868 (1996).

3. Anisimov, V. I., Zaanen, J. & Andersen, O. K. Band theory and Mott insulators: Hubbard U instead of Stoner I. *Phys*. *Rev*. *B* **44**, 943–954 (1991).

4. Monkhorst, H. J. & Pack, J. D. Special points for Brillouin-zone integrations. *Phys*. *Rev*. *B* **13**, 5188–5192 (1976).

5. Cococcioni, M. & De Gironcoli, S. Linear response approach to the calculation of the effective interaction parameters in the LDA+U method. *Phys*. *Rev*. *B* **71**, 035105 (2005).

6. Stevanović, V., Lany, S., Zhang, X. & Zunger, A. Correcting density functional theory for accurate predictions of compound enthalpies of formation : Fitted elemental-phase reference energies. *Phys*. *Rev*. *B* **85**, 115104, (2012).
